# Health Care Provider Knowledge Regarding Alpha-gal Syndrome — United States, March–May 2022

**DOI:** 10.15585/mmwr.mm7230a1

**Published:** 2023-07-28

**Authors:** Ann Carpenter, Naomi A. Drexler, David W. McCormick, Julie M. Thompson, Gilbert Kersh, Scott P. Commins, Johanna S. Salzer

**Affiliations:** ^1^Epidemic Intelligence Service, CDC; ^2^Division of Vector-Borne Diseases, National Center for Emerging and Zoonotic Infectious Diseases, CDC; ^3^Division of Rheumatology, Allergy, and Immunology, Department of Medicine, School of Medicine, University of North Carolina at Chapel Hill, Chapel Hill, North Carolina.

**Summary**

**What is already known about this topic?**

Alpha-gal syndrome (AGS) is an emerging, tick bite–associated allergic condition characterized by a hypersensitivity to an oligosaccharide found in most mammalian meat and products derived from it. Symptoms can be life-threatening and can include anaphylaxis. Cases are increasing, although patients report limited health care provider (HCP) awareness of AGS.

**What is added by this report?**

HCP respondents (N = 1,500) to a nationwide survey had limited AGS knowledge: 42% were not aware of AGS, and another 35% were not confident in their ability to diagnose or manage AGS patients.

**What are the implications for public health practice?**

Limited HCP knowledge about AGS is concerning, especially because the number of suspected cases is increasing, and the range of the tick primarily associated with this condition is expected to expand. Improved HCP education might facilitate a rapid diagnosis of AGS, improve patient care, and support public health understanding of this emerging condition.

## Abstract

Alpha-gal syndrome (AGS) is an emerging, tick bite–associated immunoglobulin E–mediated allergic condition characterized by a reaction to the oligosaccharide galactose-alpha-1,3-galactose (alpha-gal), which is found in mammalian meat and products derived from mammals, including milk, other dairy products, and some pharmaceutical products. Symptoms range from mild (e.g., a rash or gastrointestinal upset) to severe (anaphylaxis); onset typically occurs ≥2 hours after exposure to alpha-gal. No treatment or cure is currently available. Despite the potential life-threating reactions associated with AGS, most patients perceive that health care providers (HCPs) have little or no knowledge of AGS. A U.S. web-based survey of 1,500 HCPs revealed limited knowledge of AGS, identified areas for continuing medical education, and described self-reported diagnostic and management practices. Overall, 42% of surveyed HCPs had never heard of AGS, and among those who had, fewer than one third knew how to diagnose the condition. Two thirds of respondents indicated that guidelines for the diagnosis and management of AGS would be useful clinical resources. Limited awareness and knowledge of AGS among HCPs likely contributes to underdiagnosis of this condition and inadequate patient management, and underestimates of the number of AGS patients in the United States, which currently relies on laboratory testing data alone.

## Introduction

Alpha-gal syndrome (AGS) is an emerging, tick bite–associated, immunoglobulin E (IgE)–mediated allergic condition characterized by a reaction to galactose-alpha-1,3-galactose (alpha-gal), a sugar molecule found in most nonprimate mammals. Evidence suggests that the reaction is primarily associated with the bite of the lone star tick (*Ambylomma americanum*) in the United States. Cases are most prevalent in the southern, midwestern, and mid-Atlantic United States, overlapping the range of the lone star tick (*1*–*3*). No treatment or cure is currently available. Despite the potential life-threatening reactions associated with AGS, patients perceive that health care providers (HCPs) have little or no knowledge of AGS (*4*). Data from a nationwide, web-based survey of HCPs in the United States (DocStyles, Spring 2022), administered by Porter Novelli Public Services, were analyzed to determine HCP knowledge relating to the diagnosis and management of AGS.

## Methods

HCPs were identified from the SERMO Global Medical Panel, a physician networking platform with an opt-in, verified panel of medical professionals who receive an honorarium for participating in market research surveys. Panelists were verified using a double opt-in sign up process with telephone confirmation at their place of work.* SERMO identified a random sample of eligible providers from its main database and distributed an electronic invitation to participate in the study, including a link to the web-based survey.^†^ The minimum number of respondents, or survey quota, was set to reach 1,500 primary care practitioners.^§^ Respondents were providers who actively saw patients; worked in an individual, group, or hospital practice; and had practiced for >3 years.

The analysis was limited to family practitioners, general practitioners, internists, pediatricians, nurse practitioners (NPs), and physician assistants (PAs). Frequencies and percentages were calculated, and Pearson chi-square tests were used to compare categorical variables, using SAS software (version 9.4; SAS Institute). 

To assess multifactorial knowledge, a composite knowledge score was calculated for all respondents with a maximum score of 3; one point was awarded for each correct answer to the following three topics: 1) how AGS is acquired, 2) appropriate diagnosis of AGS, and 3) counseling of patients with AGS. Scores ranged from 0 (no answers correct) to 3 (all answers correct). This activity was reviewed by CDC and was conducted consistent with applicable federal law and CDC policy.^¶^

## Results

A total of 1,500 respondents completed the survey, including 1,000 primary care physicians, 250 pediatricians, and 250 PAs and NPs. Overall, 974 (65%) respondents worked in a group outpatient practice or clinic, approximately one third worked in an individual outpatient practice (235; 16%), or in an inpatient practice or a hospital (291; 19%). The largest percentage of respondents worked in the U.S. Census Bureau South Region** (472; 32%), followed by the Northeast Region (377; 25%), and the Midwest Region (337; 22%); approximately one fifth worked in the West Region (314; 21%).

Overall, 635 (42%) respondents had not heard of AGS, and another 530 (35%) reported that they were “not too confident” about their ability to diagnose or manage patients with AGS (Table 1). Only 74 (5%) felt “very confident” in their ability. Among 865 (58%) respondents who were aware of AGS, 674 (78%) had not made a diagnosis of AGS in the previous year; 136 (16%) diagnosed or managed one to five patients, and 55 (6%) diagnosed or managed more than five patients.

**TABLE 1:** Survey questions and responses by health care providers regarding their practice characteristics and knowledge about alpha-gal syndrome (N = 1,500) — Spring DocStyles survey,* United States, March–May 2022

Survey question (total no. of responses)	No. (%)	95% CI
**Where do you practice? (1,500)**		
Group outpatient clinic or practice	974 (65.9)	63.5–68.3
Individual outpatient practice	235 (15.9)	14.1–17.8
Inpatient practice or hospital	291 (18.2)	16.3–20.2
**Where is your practice located? (by U.S. Census Bureau region, 1,500)**		
South	472 (31.5)	29.1–33.9
Northeast	377 (25.1)	22.9–27.3
Midwest	337 (22.5)	20.4–24.6
West	314 (20.9)	18.8–23.0
**How confident are you in your ability to diagnose and manage patients with AGS? (1,500)**		
Very confident	74 (4.9)	3.8–6.0
Somewhat confident	261 (17.4)	15.5–19.3
Not too confident	530 (35.3)	32.9–33.7
I have not heard of this condition	635 (42.3)	39.8–44.8
**You have diagnosed a patient with AGS. Which of the following topics would you counsel them on?^†^ (865)**		
Tick bite prevention	31 (3.6)	2.4–4.8
Eliminating red meat from their diet	148 (17.1)	15.0–19.6
Caution with new vaccines or medications	60 (6.9)	5.2–8.6
Recognizing and managing anaphylaxis	124 (14.3)	12.0–16.6
All of the above^§^	502 (58.0)	54.7–61.3
**Following a detailed patient exam, which of the following tests would you order to confirm an AGS diagnosis?^†^ Select all that apply. (865)**		
sIgE to alpha-gal^§^	252 (29.1)	26.1–32.1
Allergy skin test	122 (14.1)	11.8–16.4
PCR	107 (12.4)	10.2-14.6
IgG to alpha-gal	191 (22.1)	19.3–24.9
Not sure	416 (48.1)	44.8–51.4
**How does a patient get AGS?^†^ (865)**		
From a tick bite^§^	285 (33.0)	29.9–36.1
Genetic predisposition	54 (6.2)	4.6–7.8
Immune complex–mediated	90 (10.4)	8.4–12.4
Eating too much red meat	39 (4.5)	3.1–5.9
The cause is not yet known	125 (14.5)	12.6–16.9
Don’t know	272 (31.5)	28.4–34.6
**In the past 12 months, how many of your patients reported a recent exposure to ticks? (865)**		
0	142 (16.4)	13.9–18.9
1–5	343 (39.7)	36.4–43.0
6–19	242 (28.0)	25.0–31.0
20–100	125 (14.5)	12.2–16.9
>100	13 (1.5)	0.1–2.3
**In the past 12 months, how many patients have you diagnosed or managed with AGS? (865)**		
0	674 (77.9)	75.1–80.7
1–5	136 (15.7)	13.3–18.1
>5	55 (6.4)	4.7–8.0
6–19	44 (5.1)	3.6–6.6
20–100	8 (0.9)	0–2.0
>100	3 (0.4)	0–1.0
**What additional resources would be helpful in treating and managing patients with AGS? Select all that apply*.* (865)**		
Online training modules	708 (47.2)	43.9–50.5
CDC guidelines on diagnosis of AGS	955 (63.7)	60.5–66.9
CDC guidelines on management of AGS	982 (65.5)	62.3–68.7
List of products containing alpha-gal	620 (41.3)	38.0–44.6
Website content for health care providers	807 (53.8)	50.5–57.1
No additional resources are needed	84 (5.6)	4.1–7.1

**Abbreviations:** AGS = alpha-gal syndrome; PCR = polymerase chain reaction; sIgE = alpha-gal–specific serum IgE antibody.

* Administered by Porter Novelli.

^†^ Evaluated together to generate a composite knowledge score.

^§^ Correct response.

Among all respondents who were aware of AGS, 416 (48%) reported that they did not know the correct diagnostic tests to order. One third of respondents (285; 33%) correctly reported that patients develop AGS after a tick bite, and approximately one third (272; 32%) reported not knowing how it was acquired. More than one half of the respondents (502; 58%) correctly identified topics on which to counsel AGS patients, such as tick bite prevention, eliminating red meat from their diet, exercising caution when receiving new medications and vaccines, and recognizing and managing anaphylaxis. Overall, 64% and 66% of respondents indicated that guidelines for the diagnosis and management of AGS, respectively, would be helpful clinical resources.

Among the 865 survey respondents who had heard of AGS, only 42 (5%; 95% CI = 3.1%–5.9%) correctly answered all three questions related to etiology, testing, and patient counseling (Table 2). Knowledge scores were higher among pediatricians, 12.3% of whom correctly answered all three questions, than among internists (4.2%), family practitioners (3.7%), PAs (2.6%), and NPs (0%). Knowledge scores were similar across U.S. Census Bureau regions (p = 0.44), and number of years in practice was not significantly associated with provider knowledge scores. There was an inverse relationship in knowledge scores and the number of AGS cases that HCPs reported they had diagnosed and managed (Table 2). 

**TABLE 2:** Knowledge about alpha-gal syndrome among health care providers, overall and by region and provider characteristics (N = 865) — Spring DocStyles survey,* United States, March–May 2022

Characteristic	No. (%) of questions answered correctly				Mean (SD)	Total	Chi-square, p-value
	0	1	2	3			
Overall composite knowledge score	213 (24.62)	417 (48.21)	193 (22.31)	42 (4.86)	1.07 (0.81)	865	—
**U.S. Census Bureau region^†^**							
Northeast	48 (24.49)	97 (49.49)	44 (22.45)	7 (3.57)	1.05 (0.78)	196	0.15
Midwest	50 (24.27)	99 (48.06)	47 (22.82)	10 (4.85)	1.08 (0.81)	206	0.31
South	64 (21.99)	135 (46.39)	74 (25.43)	18 (6.19)	1.16 (0.84)	291	Ref
West	51 (29.65)	86 (50.00)	28 (16.28)	7 (4.07)	0.95 (0.79)	172	<0.05
**Total**	**213**	**417 **	**193 **	**42 **	**1.07 (0.81)**	**865**	** 0.44**
**No. of yrs in practice**							
<5	18 (16.8)	62 (57.9)	22 (20.6)	5 (4.7)	1.13 (0.74)	107 (12.4)	0.57
6–10	51 (24.3)	100 (47.6)	50 (23.8)	9 (4.3)	1.08 (0.81)	210 (24.3)	0.19
11–15	52 (29.9)	80 (46.0)	37 (21.3)	5 (2.9)	0.97 (0.79)	174 (20.1)	<0.05
16–20	42 (33.1)	54 (42.5)	27 (21.3)	4 (3.2)	0.94 (0.82)	127 (14.7)	<0.05
>20	50 (20.2)	121 (49.0)	57 (23.1)	19 (7.7)	1.18 (0.84)	247 (28.6)	Ref
**Total**	**213**	**417**	**193**	**42**	**1.07 (0.81)**	**865**	**0.06**
**Provider type**							
Pediatrician	28 (21.5)	49 (37.7)	37 (28.5)	16 (12.3)	1.32 (0.95)	130 (15.0)	Ref
FP	68 (25.0)	137 (50.5)	57 (21.0)	10 (3.7)	1.03 (0.78)	272 (31.5)	<0.01
Internist	87 (26.4)	161 (48.8)	68 (20.6)	14 (4.2)	1.03 (0.80)	330 (38.2)	<0.01
NP	12 (21.1)	31 (54.4)	14 (24.6)	0 (—)	1.04 (0.68)	57 (6.6)	0.02
PA	18 (23.7)	39 (51.3)	17 (22.4)	2 (2.6)	1.04 (0.76)	76 (8.8)	0.02
**Total**	**213**	**417**	**193**	**42**	**1.07 (0.81)**	**865**	**<0.05**
**No. of cases diagnosed or no. of patients managed**							
0	154 (22.9)	346 (51.3)	148 (22.0)	26 (3.9)	1.07 (0.77)	674 (77.9)	0.05
1–5	29 (21.3)	58 (42.7)	38 (27.9)	11 (8.1)	1.22 (0.88)	136 (15.7)	Ref
6–19	22 (50.0)	12 (27.3)	5 (11.4)	5 (11.4)	0.84 (1.03)	44 (5.1)	0.03
20–100	6 (75.0)	0 (—)	2 (25.0)	0 (—)	0.50 (0.93)	8 (0.9)	0.06
>100	2 (66.7)	1 (33.3)	0 (—)	0 (—)	0.33 (0.58)	3 (0.4)	0.11
**Total**	**213**	**417**	**193**	**42**	**1.07 (0.81)**	**865**	**<0.05**

**Abbreviations**: FP = family practitioner; NP = nurse practitioner; PA = physician assistant; Ref = referent group.

* Administered by Porter Novelli.

^†^ https://www2.census.gov/geo/pdfs/maps-data/maps/reference/us_regdiv.pdf

## Discussion

This analysis indicated a low level of knowledge among U.S. HCPs regarding the diagnosis and management of AGS, with 78% of providers having little to no knowledge of AGS. Previous assessments of AGS knowledge among HCPs in the United States were limited to small studies within individual jurisdictions but found similar patterns of an overall lack of knowledge among those surveyed (*5*,*6*).

Few HCPs reported diagnosing AGS or managing patients with AGS within the previous year, despite an annual increase in the number of tests performed and suspected AGS cases identified nationally and the number of persons who received positive test results increasing from 13,371 in 2017 to 18,885 in 2021^††^ (*1*,*3*). Provider knowledge of AGS etiology, testing, and patient counseling decreased as the number of patients they reported diagnosing or managing with AGS increased. This inverse association suggests that some HCPs might be incorrectly diagnosing AGS, possibly on the basis of symptoms or testing alone, and subsequently recommending dietary modifications where none are warranted. This limited provider knowledge might also lead to delayed or missed diagnosis and incorrect patient management. A growing number of resources are available for HCPs seeking additional education related to the evaluation, diagnosis, and management of patients with AGS (*7**,**8*). Diagnosis of AGS requires careful elicitation of a history in a patient with compatible symptoms, and diagnostic testing for alpha-gal–specific IgE antibodies (≥0.1 kU/L is considered a positive test result) (*8*). A 2015 study found that approximately one fifth (21%) of patients received a diagnosis within their first year of signs and symptoms, whereas the remaining 79% received a diagnosis in an average of 7.1 years (*9*). Repeated visits to HCPs and referrals to specialists might be necessary for patients to receive a proper diagnosis and care, creating a disadvantage to those patients who face challenges seeking health care in general or who lack access to specialty practitioners, such as allergists.

### Limitations

The findings in this report are subject to at least two limitations. First, the findings might not be generalizable to all practicing HCPs in the United States since respondents were part of a provider panel. Second, providers might have interpreted response options differently. For example, when asked about how a patient acquires AGS, one response option was “the cause is not yet known.” Although tick bites have been widely recognized as triggering the hypersensitivity to alpha-gal (*2*), and “tick bites” was considered the correct response, the detailed immunologic aspects of the tick bite etiology of AGS are still being investigated. These possible differences in interpretation, as well as the nature of self-reporting, might have contributed to misclassification of responses as being correct or incorrect.

### Implications for Public Health Practice

Considering the recent description of a continued increase in the number of persons receiving positive alpha-gal–specific IgE (sIgE) antibody test results, growing numbers of suspected AGS cases (*3*), and expanding North American ranges of the lone star tick (*10*), the knowledge gap found in this survey of HCPs is concerning. Currently, AGS is not a nationally notifiable condition, and understanding epidemiologic trends relies on laboratory-based surveillance (*1*,*3*). The lack of HCP knowledge of AGS is likely to lead to undertesting, further hampering knowledge of the national prevalence of AGS.^§§^ Increased HCP education and awareness of AGS are needed to hasten and improve the accuracy of AGS diagnoses, patient care, and the understanding of the epidemiology of this emerging condition.

## Footnotes

*
https://styles.porternovelli.com/docstyles

† Panelists were verified using a double opt-in sign-up process with telephone confirmation at place of work.

§ A total of 1,000 family or general practitioners and internists, 250 pediatricians, and 250 mid-level health care providers (nurse practitioners and physician assistants).

¶ 45 C.F.R. part 46, 21 C.F.R. part 56; 42 U.S.C. Sect. 241(d); 5 U.S.C. Sect. 552a; 44 U.S.C. Sect. 3501 et seq.

**
https://www2.census.gov/geo/pdfs/maps-data/maps/reference/us_regdiv.pdf

†† The national standardized case definition accepted in 2021 by the Council of State and Territorial Epidemiologists defined a confirmed case of AGS as being in a person who met the clinical criteria and confirmatory laboratory evidence (serum or plasma sIgE specific to alpha-gal ≥0.1 IU/mL or ≥0.1 kU/L). A suspected case of AGS was defined as being in a person who had confirmatory laboratory evidence with no clinical information available. https://ndc.services.cdc.gov/case-definitions/alpha-gal-syndrome-ags/

§§
https://www.cdc.gov/ticks/alpha-gal/index.html
